# Exploring Salivary Thiocyanate as a Novel Biomarker of Physical Activity Response

**DOI:** 10.3390/molecules30112476

**Published:** 2025-06-05

**Authors:** Christoforos Chrimatopoulos, Kalliopi Papadopoulou, Theodora Tsilouli, Vasilios Sakkas

**Affiliations:** Department of Chemistry, School of Sciences, University of Ioannina, 45110 Ioannina, Greece; c.chrimatopoulos@uoi.gr (C.C.); pch01468@uoi.gr (K.P.); pch01456@uoi.gr (T.T.)

**Keywords:** sports physiology, physical exercise, diverse cohort of athletes, non-invasive saliva sampling, photometric analysis, exercise-induced biomarker

## Abstract

Salivary thiocyanate (SCN^−^) has long been recognized for its role in mucosal defense and antioxidant function, yet its behavior during physical activity remains unexplored. This study aimed to investigate salivary thiocyanate as a novel salivary biomarker responsive to exercise. A standard Vis–photometric method (thiocyanatoiron-complex formation) was utilized and validated for the rapid quantification of thiocyanate in saliva. The method was applied to two experimental setups: (i) a controlled treadmill protocol involving incremental running intensities (20%, 60%, and 90% VO_2_max-mL/kg/min), and (ii) field sampling of athletes from various sports before and after their typical training sessions, managing a total of 162 athletes. This work is the first to quantitatively investigate thiocyanate as an exercise-induced salivary biomarker, validated through both controlled and real-world settings. Additionally, subgroup analysis across sex and smoking status revealed inter-individual variation in salivary SCN^−^ profiles. Across both setups, during controlled exercise intensity increment and typical training routine, thiocyanate concentrations consistently decreased in response to physical exertion. These results were statistically significant and reflected in both male and female athletes. This is the very first study that determines salivary SCN^−^ during any kind of physical exercise and opens new pathways for non-invasive sampling and for monitoring physiological stress and immune response in athletic populations.

## 1. Introduction

Physical exercise profoundly enhances both mental well-being and physical health, reducing stress, promoting cardiovascular function, and fostering overall vitality in humans [1]. In recent years, an increasing number of governments have launched campaigns to promote sports participation and encourage physical fitness through regular exercise, recognizing its significant benefits for public health [2,3]. However, the insufficient athletic culture of amateur athletes, and even more so, the excessive training routines of professional athletes to improve their performance and achieve the best possible outcome [4,5], may lead to overtraining, with serious health consequences [6]. Phenomena of excessive fatigue and chronic injuries are observed [7,8] because of pushing the athlete’s body to the limit.

In this way, various modern methods (heart rate monitors, biomechanical analyses, metabolomic assessments, etc. [8]) have been developed to monitor and avoid overtraining while achieving optimal performance for the athletes, a trend that has arisen in recent years [9,10]. In the last few years, metabolomics has met traditional physical assessments [11], highlighting the value of metabolite monitoring. Thus, the field of metabolomics that focuses on biochemical alterations during exercise, named sportomics [12,13], may assist sports teams and professional athletes, unraveling the metabolic signatures of the human body.

Biological fluids, being the metabolic window of the body [14,15], are the ultimate matrix to detect alterations of biomolecules during physical exercise. Recent studies utilized blood [8], serum [16], plasma [17], urine [18,19], sweat [20], or saliva [7,21] to explore biochemical alterations resulting from different types of sport activities [22], such as football [23], basketball [24], kickboxing [25], marathon [26], rugby [27], etc. Blood, serum, and plasma contain a variety of biomarkers in high concentrations [8]; however, its collection is considered complex and invasive [9], and it carries a risk of infection/cross-contamination during blood collection [28]. On the other hand, saliva, sharing many common components with blood, even at lower concentrations [29,30], is a promising tool for metabolic studies owing to safe, easy, and non-invasive sampling [9,28,31], using cotton swabs or just by passive drooling in a sterilized container [31,32]. Saliva, serving as a diagnostic tool, has been utilized to study a wide spectrum of diseases, including but not limited to Alzheimer’s [33,34], cancer [35] (pancreatic [36], oral [37], lung [38], etc.), and periodontal disease [39,40], while in the last couple of years, saliva has proven useful for the assessment of the metabolic impact of physical exercise [41,42]. Indeed, some scientific approaches have shown that salivary lactate is an index of fatigue that increases especially with exercise [31,43,44,45].

Saliva, being the product of oral glands [28,31,46], consists of 99% water and 1% organic and inorganic compounds [46,47], housing proteins [48], enzymes [49], amino [50] and fatty acids [51], hormones [4,52,53] (cortisol, testosterone, etc.), and nucleic acids [54,55] (DNA, mRNA, etc.). However, the composition of saliva is strongly related to everyday factors and endogenous variations such as age [56,57], sex [58], smoking [59,60], and medications [61,62].

Thiocyanates, which are well known for their antibacterial action [63,64], are present in human saliva at concentrations ranging from 0.5 to 3 mM, making saliva the biofluid with the highest thiocyanate content [65]. This antibacterial action is attributed to the sequential formation of oxidation products: OSCN^−^, HOSCN, O_2_SCN^−^, and O_3_SCN^−^, resulting from the hydrogen peroxide-mediated oxidation of thiocyanate [66,67,68]. Despite the well-established role of thiocyanates as biomarkers for cigarette smoking [69,70,71], research investigating their correlation with physical exercise remains extremely limited. Only a handful of studies have explored the changes in salivary thiocyanate levels during exercise [44,72], highlighting a significant gap in the literature.

Given the significance of thiocyanate in both health and environmental settings, the development of reliable techniques for its detection is essential. A range of methodologies has been proposed, including chromatography [73], Fourier-transform infrared spectroscopy (FTIR) [44,74,75], amperometry [76], capillary electrophoresis [72], and other methodologies [71,77]. However, the UV–vis spectrophotometric technique is the most widely used for the determination of thiocyanates in different matrices, including saliva [78].

Herein, this study aims to investigate variations in salivary thiocyanate concentration during physical exercise and evaluate its potential as a biomarker for endurance performance. By employing a simple and user-friendly Vis–photometric technique, validating and examining its analytical assumptions, this research provides a quantitative assessment of thiocyanate fluctuations in response to different exercise intensities, contributing to a deeper understanding of its physiological role in exercise adaptation.

## 2. Results and Discussion

### 2.1. Method Validation

**Figure 1:**
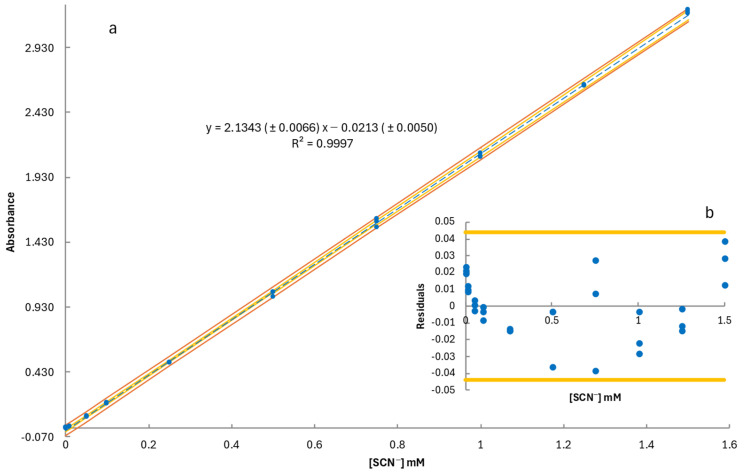
(**a**) Calibration curve for thiocyanate (blue dashed line) with 95% confidence (yellow lines) and prediction (orange lines) intervals and (**b**) residuals plot showing deviation limits.

The calibration curve for thiocyanate determination was expressed as y = b (±*S_b_*) x + α (±*S_α_*), where b represents the slope (*S_b_*: standard error of the slope) and α the intercept (*S_α_*: standard error of the intercept). Standard SCN^−^ solutions were prepared in artificial saliva, covering a concentration range of 0.01 to 1.5 mM across ten concentration levels, with three replicates per level. The calibration curve, along with confidence and prediction intervals, is presented in Figure 1a. The residuals plot in Figure 1b confirmed the absence of a trumpet-shaped pattern at higher concentrations, indicating homoscedasticity across the working range. Additionally, the horizontal lines (±*t* (0.05, [conc. levels] − 2) × *S_y/x_*) in the residual plot established deviation limits for individual data points, verifying that no outliers were present, thereby ensuring the robustness of the calibration model [79,80].

The statistical significance of the regression model was further validated through an analysis of variance (ANOVA) (Table 1). The computed *F*-value (105,079.18) significantly exceeded the critical *F_crit_* value (4.35), with an exceptionally low *p*-value (1.35 × 10^−51^), confirming the strong linear relationship between concentration and absorbance [79]. The high *F*-value, minimal residual error, and strong coefficient of determination (R^2^) demonstrate the reliability of the model for accurate thiocyanate quantification in saliva samples.

The developed method was further validated by determining the limits of detection (LOD) and quantification (LOQ). These values were calculated from the calibration curve using the standard equations LOD = 3 × s/b and LOQ = 10 × s/b, where b is the slope of the calibration curve (2.1343), and s represents the standard deviation of the response. The standard deviation was estimated using three approaches: (i) the residual standard deviation of the regression (*S_y/x_*), (ii) the standard deviation of the intercept (*S_α_*), and (iii) the standard deviation of blank measurements. The lowest theoretical LOQ value, which was experimentally confirmed, was indicated as the final limit of quantification.

The analytical performance of the method is summarized in Table 2. The method demonstrated high sensitivity, with an LOD of 0.004 mM and an LOQ of 0.01 mM, enabling the reliable detection of thiocyanate even at low concentrations. The working range of the method spans from 0.01 to 1.5 mM, covering the expected thiocyanate concentrations in saliva samples collected before and after exercise.

### 2.2. Thiocyanate Response to Graded Exercise

**Figure 2:**
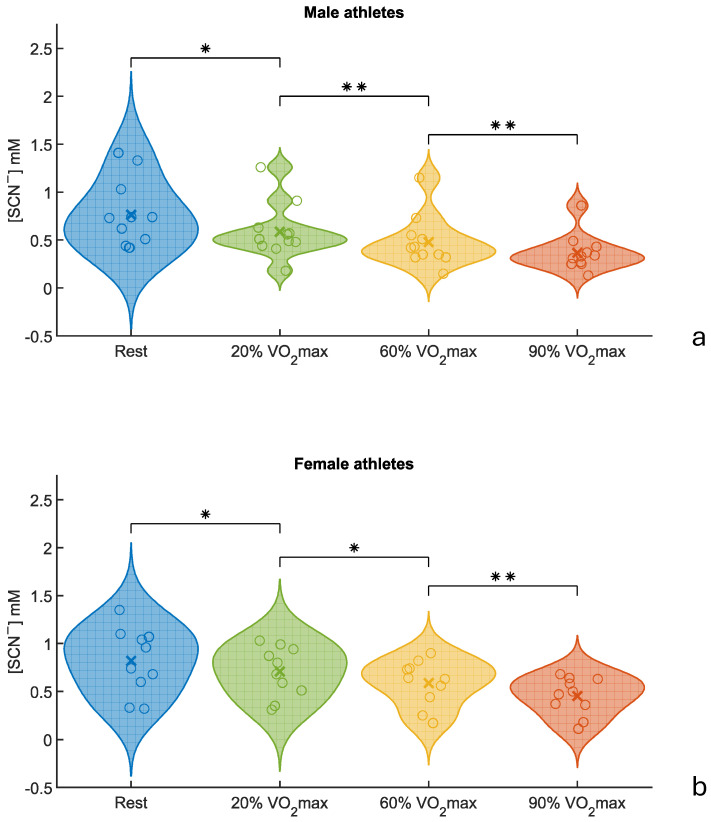
Violin plots of thiocyanate variations and mean values (x symbol) in saliva, during an increase in exercise intensity: (**a**) male athletes and (**b**) female athletes. * *p*-adjusted < 0.05, ** *p*-adjusted < 0.01.

To investigate the effect of increasing exercise intensity on salivary thiocyanate (SCN^−^) levels, saliva samples were collected from 21 athletes (11 males and 10 females) at four time points: at rest and after running 1 km at 20%, 60%, and 90% of their VO_2_max (mL/kg/min) on a treadmill. The violin plots (Figure 2) illustrate a consistent decrease in SCN^−^ concentrations across all exercise intensities in both male and female participants, with the highest values at rest and the lowest following the most intense exertion. Despite individual variability, the overall trend was clear: the salivary thiocyanate levels declined progressively as exercise intensity increased.

A repeated-measures ANOVA confirmed that exercise intensity had a statistically significant effect on SCN^−^ levels in both males (*p* = 0.014) and females (*p* = 0.024). Bonferroni-adjusted post hoc comparisons revealed that, in males, thiocyanate concentrations significantly decreased from rest to 20% VO_2_max (*p*-adjusted < 0.05), with even greater significance observed between the 20 and 60% and the 60 and 90% VO_2_max stages (both *p*-adjusted < 0.01). In females, significant differences were found between rest and 20% VO_2_max, as well as between 20 and 60% VO_2_max (both *p*-adjusted < 0.05), while the final stage (60–90%) met the stricter threshold (*p*-adjusted < 0.01). These results are summarized in Table 3.

These findings are in strong agreement with those of a previous study [44], where ATR-FTIR spectroscopy demonstrated reduced IR signal intensity in the SCN^−^ band (2050–2060 cm^−1^) as exercise intensity increased. The alignment between spectroscopic and photometric results reinforces the validity of thiocyanate as a responsive salivary biomarker of physical exertion. In addition, a previous study demonstrated a decrease in salivary SCN^−^ concentrations, measured via capillary electrophoresis, following running sessions [72].

Overall, this controlled study provides strong evidence that the salivary thiocyanate concentration is inversely related to exercise intensity, offering a reliable and non-invasive means of tracking physiological stress during physical activity. A limitation of this part is the relatively small size of the controlled cohort (*n* = 21), which may reduce the statistical power. While significant trends were observed, future studies with a larger number of both male and female participants in this controlled setup are necessary. In addition, further research involving larger and more diverse cohorts is required to confirm these promising results and better account for inter-individual variability (see Section 2.3).

### 2.3. Monitoring Salivary Thiocyanate During Real-World Training Sessions

As a logical next step, the investigation was extended to real-world training environments by monitoring athletes from diverse sports disciplines before and after their typical training sessions. This broader context allowed for assessing the biomarker’s behavior across different exercise modalities and its practical applicability in sports science.

Investigating salivary thiocyanate as a biomarker in a diverse athletic cohort reduces individual variability and enhances statistical power. To assess thiocyanate dynamics, saliva samples were collected from each athlete before and immediately after training. The participants (141 in total) were categorized into four subgroups based on gender and smoking habits: 18 male smokers, 59 male non-smokers, 12 female smokers, and 52 female non-smokers. Smoking, a major and well-documented contributor to elevated salivary SCN^−^ levels, was specifically recorded and analyzed as a subgroup due to its strong and direct impact. While specific dietary sources of thiocyanate (e.g., cruciferous vegetables and almonds) were not tracked, the use of paired pre- and post-exercise comparisons helped mitigate the influence of background factors.

**Figure 3:**
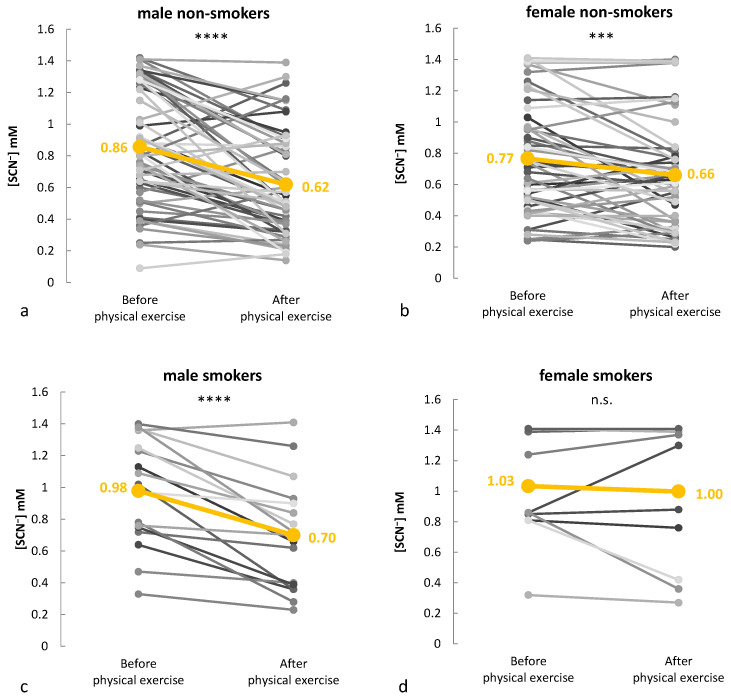
Individual alterations (grey-scale points) in salivary SCN^−^ levels before and after exercise in (**a**) male non-smokers (*n* = 59), (**b**) female non-smokers (*n* = 52), (**c**) male smokers (*n* = 18), and (**d**) female smokers (*n* = 12). The yellow lines indicate the mean values for each group. Statistical significance for paired pre/post comparisons is represented as n.s. (not significant), *** *p* < 0.001, **** *p* < 0.0001.

In Figure 3, individual changes in thiocyanate levels are illustrated, with mean values before and after exercise highlighted. In male smokers, the average thiocyanate concentration dropped from 0.98 mM to 0.70 mM, while male non-smokers showed a decrease from 0.86 mM to 0.62 mM. Likewise, the thiocyanate level of female non-smokers decreased from 0.77 mM to 0.66 mM. In contrast, the female smokers showed no significant change, with their mean concentration remaining stable between 1.03 and 1.00 mM.

A notable sex-related difference was also observed, with males exhibiting a greater reduction in thiocyanate levels post-exercise compared to females. Male non-smokers showed a mean decrease of 0.24 mM, while female non-smokers experienced a more moderate decline of 0.11 mM. Indeed, in the aforementioned controlled setup experiment, the male group exhibited a greater overall decline in thiocyanate levels compared to the female group, suggesting possible sex-related physiological influences such as differences in respiratory adjustments [81] or even in salivary flow rate and gland size [82] between genders. A previous study has highlighted sex-based differences in metabolic responses to running, demonstrating that males tend to rely more on carbohydrate utilization, whereas females exhibit a greater dependence on fat as a primary energy substrate during exercise [83].

A paired *t*-test was performed within each group to compare pre- and post-exercise thiocyanate levels, ensuring intra-individual variations were accounted for. The analysis revealed a significant decrease in thiocyanate concentration in the male smokers (*p* < 0.0001), male non-smokers (*p* < 0.0001), and female non-smokers (*p* < 0.001), supporting the role of thiocyanate as an exercise-responsive biomarker. Interestingly, no statistically significant change was observed in the female smokers, possibly due to the smaller sample size and higher baseline thiocyanate levels in smokers, which may have masked potential exercise-induced changes. In line with previous findings, both male and female smokers had higher initial thiocyanate concentrations than their non-smoking counterparts, reflecting the accumulation of thiocyanate from tobacco exposure [84].

Although our design captured immediate pre- and post-exercise SCN^−^ concentrations, the temporal kinetics of SCN^−^ recovery were not examined. Understanding how quickly SCN^−^ levels return to baseline post-exercise—and whether this is influenced by factors such as exercise intensity, sex, or metabolic status—represents a valuable direction for future research.

Overall, the findings reinforce the potential of thiocyanate as a salivary biomarker for exercise-induced physiological changes, while also highlighting sex- and smoking-related variations in thiocyanate metabolism.

### 2.4. Biochemical Mechanisms of Exercise-Induced Thiocyanate Reduction

Thiocyanate (SCN^−^), a pseudohalide thiolate, is one of the most abundant ions in saliva, with concentrations ranging from 0.5 to 3 mM in non-smokers and up to 6 mM in heavy smokers due to dietary and environmental exposure [65,84]. SCN^−^ plays a crucial role in the lactoperoxidase (LPO) system, which contributes to innate immune defense by generating antimicrobial molecules such as hypothiocyanite (OSCN^−^) in the presence of hydrogen peroxide (H_2_O_2_) [85,86].

During physical exercise, biochemical interactions involving SCN^−^ and peroxidase activity [87] influence its concentration. Studies have shown that salivary peroxidase activity increases immediately after intense exercise and gradually returns to baseline within an hour [88,89]. The decline in salivary SCN^−^ levels during exercise is largely attributed to its oxidative conversion via the LPO system, where H_2_O_2_ reacts with SCN^−^ to form antimicrobial compounds [66].

This process is further stimulated by salivary lactate, which rises significantly during high-intensity exercise due to anaerobic metabolism [41]. Lactate metabolism enhances H_2_O_2_ production [90,91] through a flavin-dependent lactate oxidase pathway [92], thereby accelerating the oxidation of SCN^−^. Lactate metabolism interplays with salivary peroxidase activity [66,93], increasing H_2_O_2_ production during exercise. This accelerates SCN^−^ oxidation, leading to its reduced concentration in saliva. In addition, the strong correlation between salivary lactate levels and thiocyanate IR bands suggests a direct biochemical link among exercise intensity, lactate metabolism, and SCN^−^ reduction [67].

Although the observed decline in salivary SCN^−^ concentration post-exercise aligns with the proposed involvement of the LPO system—where elevated H_2_O_2_, potentially derived from lactate metabolism, drives SCN^−^ consumption—this mechanistic pathway was not directly assessed in this research. Future studies incorporating direct measurement of lactate, H_2_O_2_, and LPO activity could help validate this proposed mechanism.

These findings indicate that the decrease in SCN^−^ concentration during exercise is not a passive depletion but an active biochemical response to increased metabolic and enzymatic activity. This reduction may serve as a functional biomarker of exercise intensity, providing insights into physiological adaptations during physical exertion.

## 3. Materials and Methods

### 3.1. Method Development

A detailed analysis of thiocyanate ions in saliva was conducted using a well-established thiocyanatoiron (III) assay [78]. In contrast to the previous study [78], to develop and validate the method, a calibration curve was constructed using artificial saliva, ensuring a more accurate representation of the sample matrix. Thiocyanate standards ranging from 0.01 to 1.5 mM were prepared by dissolving potassium thiocyanate (KSCN, Sigma-Aldrich, St. Louis, MO, USA) in artificial saliva to create a 1.5 M stock solution, which was subsequently diluted as needed.

Artificial saliva (free of SCN^−^) was prepared following a standardized composition [71], containing 125.6 mg/L sodium chloride (NaCl, Riedel-de Haën, Seelze, Germany), 963.9 mg/L potassium chloride (KCl, Carlo Erba, Milan, Italy), 227.9 mg/L calcium chloride dihydrate (CaCl2 ⋅ 2H_2_O, Ferak Berlin, Berlin, Germany), 178 mg/L ammonium chloride (NH_4_Cl, Mallinckrodt, St. Louis, MO, USA), 336.5 mg/L sodium sulphate (Na_2_SO_4_, Merck, Darmstadt, Germany), 200 mg/L urea (CH_4_N_2_O, Carl Roth, Karlsruhe, Germany), 630.8 mg/L sodium bicarbonate (NaHCO_3_, Mallinckrodt, St. Louis, MO, USA), and 654.5 mg/L potassium dihydrogen phosphate (KH_2_PO_4_, Merck, Darmstadt, Germany), all dissolved in deionized water. For complex formation and thiocyanate detection, a 0.2 M iron(III) nitrate (Fe(NO_3_)_3_, Sigma-Aldrich, St. Louis, MO, USA) reagent was prepared in 1 M nitric acid.

In the assay, 1.0 mL of thiocyanate standard was mixed with 1.0 mL of the iron(III) reagent, allowing the reaction to occur via vortex mixing. The thiocyanatoiron(III) blood-red colored complex formed immediately, and its absorbance was measured photometrically at 458 nm using a UV–vis spectrophotometer (UV-1800 Spectrophotometer, Shimadzu, Kyoto, Japan).

### 3.2. Bioethical Approval

This study adhered to ethical guidelines and was approved by the Independent Personal Data Protection Department of the University of Ioannina (protocol number 10253/18-1-2022). Informed consent was obtained from all participants, ensuring confidentiality and the right to withdraw. Demographic and lifestyle information was collected using a structured questionnaire, which was completed together with the consent form (Supplementary Materials). They were both completed before any sampling or data collection took place. Anonymity was maintained through coded sample collection.

### 3.3. Participants

Athletes were recruited through active collaboration with sports centers and academies over six months. Engagement with local sports clubs and institutions and personal coaches facilitated the selection of a diverse and representative cohort. This study included a total of 162 athletes (88 males and 74 females) from various sports disciplines, i.e., football, basketball, pole dancing, aerial hoops, tennis, volleyball, middle-distance running, and aerobic gymnastics. Exclusion criteria included consumption of stimulant supplements, or any drugs affecting physical performance. Smoking was not included as an exclusion criterion due to its strong correlation with thiocyanate concentration in saliva; however, smoker athletes were identified and analyzed separately to account for its potential impact on the results. The anthropometric characteristics of the participants were as follows: mean ages of 24.6 ± 6.8 and 24.1 ± 4.9 years, heights of 1.82 ± 0.06 and 1.76 ± 0.05 m, and body mass index (BMI) values of 22.14 ± 1.41 and 21.02 ± 1.19 kg/m^2^ for males and females, respectively. The participants were thoroughly informed about this study’s objectives, methodology, and data confidentiality before providing consent.

### 3.4. Cohort Management and Experimental Design

**Figure 4:**
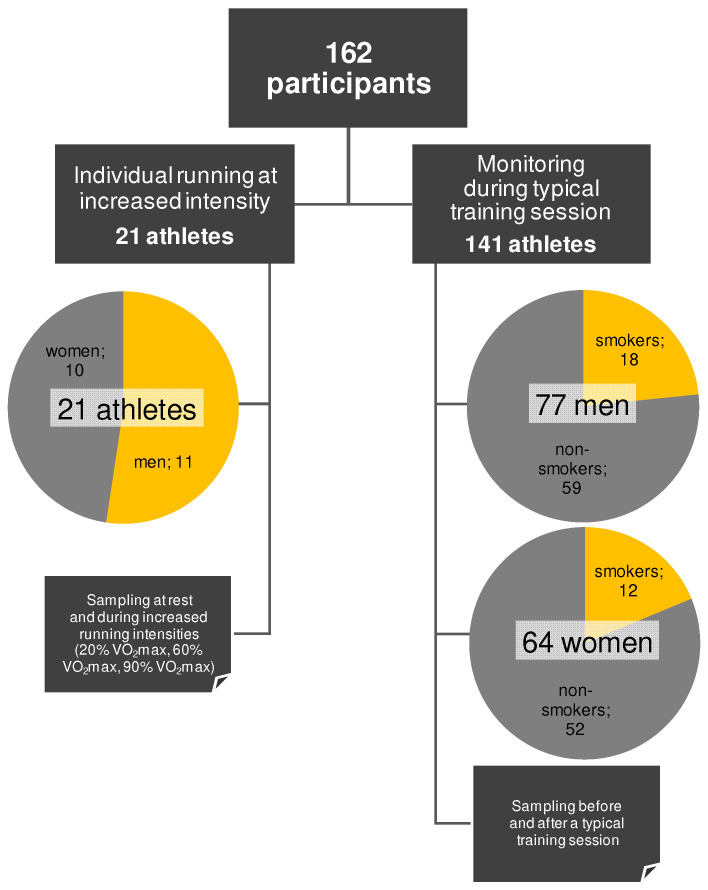
Cohort management and demographic statistics of participants.

This study was conducted in two parts. In the first part, 21 athletes (only non-smokers) ran on a treadmill individually at specific intensity, i.e., 20%, 60%, and 90% of VO_2_max (mL/kg/min), with each athlete covering 1 km at each intensity, totaling 3 km. The VO_2_max value of each athlete was measured via an ergometric test in a third-party laboratory using the Bruce treadmill protocol, a widely accepted graded exercise test designed to progressively increase workload until volitional exhaustion. Each test was performed using the same metabolic cart and treadmill model to maintain consistency across measurements. Prior to each test, equipment calibration and participant familiarization were conducted in accordance with the laboratory’s standard operating procedures. The athletes who participated in this part were instructed not to eat anything, drink any caffeinated beverages, or perform any oral hygiene for 2 h before sampling. Water consumption was not restricted. In the second part, additional sampling was performed from a larger cohort of athletes, this time monitoring the SCN^−^ on a regular training day, to validate the findings from the first part. Thus, a cohort of 141 participants provided demographic and lifestyle information, including sex, age, time since their last meal, coffee consumption, oral hygiene activity, and smoking status (Supplementary Materials). Saliva samples from the larger cohort were collected before and after their typical training sessions at their respective training facilities. Figure 4 depicts the experimental design of the whole study.

### 3.5. Saliva Collection, Preparation, and Analysis

Saliva samples were collected at rest and after running at different intensities (in the first part) or after athletes’ training session (in the second part), as described earlier. The participants were advised to drink a small amount of water prior to sampling to avoid dehydration. Sampling was performed using a sterile dental cotton swab, chewing it for a few seconds, to stimulate saliva production, and discarding it into a Salivette^®^ tube (SARSTEDT, Numbrecht, Germany). After sampling, the saliva was either analyzed immediately or stored in a refrigerator at 4 °C for no more than 4 h. To extract the saliva, the cotton swabs were centrifuged at 10,000 rpm for 15 min using a Centurion Scientific K241 centrifuge (Centurion Scientific Ltd., Chichester, WS, UK), and the supernatant was collected for analysis. Real saliva samples were analyzed following the same procedure as the standard solutions by mixing an equal amount of the supernatant saliva sample with the iron(III) nitrate reagent; the mixture was measured photometrically at 458 nm.

### 3.6. Statistical Analysis

The developed method was validated by evaluating LOD and LOQ values, accuracy, precision and uncertainty, after the analysis of the residuals’ behavior, checking for outliers, homoscedasticity, and linearity assumptions, based on previous studies [79,80].

To evaluate statistical significance in the SCN^−^ variations across exercise intensities, an analysis of variance (ANOVA) was used, followed by post-hoc tests to identify specific group differences. The Bonferroni correction was applied to control multiple comparisons and minimize false positives. In the second part of this study, a paired *t*-test was performed for further analysis. All statistical tests were conducted using Microsoft Excel 2007 (Microsoft Corporation, Redmond, WA, USA).

## 4. Conclusions

This research is among the first to comprehensively demonstrate that salivary thiocyanate levels are systematically influenced by physical exercise, proposing thiocyanate as a promising non-invasive biomarker for physical exertion. Through the development and validation of a fast, cost-effective, and accurate photometric method, thiocyanate concentrations were quantitatively measured in saliva across a large and diverse athletic population. The method was applied in both controlled treadmill experiments and real-world training environments, confirming thiocyanate’s consistent decrease in response to physical exertion. Importantly, this study involved an extensive cohort, including both male and female athletes and further categorizing them by smoking habits, allowing for a deeper understanding of inter-individual variation. This study’s novelty lies in large-scale population monitoring, marking a significant step toward the use of thiocyanate as a practical, non-invasive saliva sampling biomarker for physiological monitoring in sport and health sciences. The findings offer a foundation for future research into thiocyanate’s role in exercise-induced oxidative stress and immune modulation, and pave the way for its use in sports monitoring, personalized training, or even early signs of physiological stress or imbalance in athletes.

## Figures and Tables

**Table 1:** Regression output of thiocyanate calibration curve.

*Regression Statistics*					
Multiple R	0.999866794				
R Square	0.999733605				
Adjusted R Square	0.999724091				
Standard Error	0.018990959 ^a^				
Observations	30				
ANOVA					
	*df*	*SS*	*MS*	*F*	*Significance F*
Regression	1	37.89749192	37.89749192	105,079.1775	1.35487 × 10^−51^
Residual	28	0.010098383	0.000360657		
Total	29	37.9075903			
	*Coefficients*	*Standard Error*	*t Stat*	*p-value*	
Intercept	−0.021333701	0.004970841 ^b^	−4.291768989	0.000191323	
X Variable 1	2.134258228	0.006583982 ^c^	324.1591855	1.35487 × 10^−51^	
	*Lower 95%*	*Upper 95%*			
Intercept	−0.031516007	−0.011151395			
X Variable 1	2.120771552	2.147744903			

^a^ *S_y/x_* = residual standard deviation, ^b^ *S_α_* = intercept standard deviation, and ^c^ *S_b_* = slope standard deviation.

**Table 2:** Analytical parameters of thiocyanate quantification method (*n* = 20).

	Analytical Parameters	
**LOD (mM)**	0.004	
**LOQ (mM)**	0.01	
**Working range (mM)**	0.01–1.5	
**Uncertainty (%)**	4.51	
**Accuracy (%)**	0.1 mM	110.22
	0.7 mM	98.36
	1.25 mM	97.57
**Intra-day repeatability** **(% RSD)**	0.1 mM	3.70
	0.7 mM	0.57
	1.25 mM	0.54
**Inter-day repeatability/reproducibility** **(% RSD)**	0.1 mM	3.13
	0.7 mM	0.83
	1.25 mM	0.56

**Table 3:** ANOVA (*p*-values) and post-hoc Bonferroni results across exercise intensity groups (adjusted *p*-values < 0.05 are underlined, and those <0.01 are double-underlined).

	ANOVA	Post-Hoc Test (Bonferroni)		
		Rest–20% VO_2_max (mL/kg/min)	20–60% VO_2_max (mL/kg/min)	60–90% VO_2_max (mL/kg/min)
**Men**	0.013949	0.016197	0.001770	0.001243
**Women**	0.023837	0.009487	0.015651	0.001174

## Data Availability

The data presented in this study are available on request from the corresponding author due to privacy reasons.

## Supplementary Materials

The following supporting information can be downloaded at: https://www.mdpi.com/article/10.3390/molecules30112476/s1, File S1. Athlete Questionnaire.
