# Supplementary material for: Exploring Salivary Thiocyanate as a Novel Biomarker of Physical Activity Response
Source: Molecules. 2025 Jun 5;30(11):2476. doi: 10.3390/molecules30112476 (PMC12157199; doi:10.3390/molecules30112476)
Supplement: Supplementary file 1 [file molecules-30-02476-s001.zip › molecules-3658224-supplementary.pdf]

Athlete Questionnaire

Lab of Analytical Chemistry
Chemistry Dpt
University of Ioannina

Sample code number ___________

1. Date _____ 2. Sport _________ 3. Sport club _________

4. Name ___________________________________

5. Gender ☐ Male ☐ Female

6. Age _____ 7. Height ________ 8. Weight ________

Basic Information

9. Are you a smoker?

☐ Yes ☐ No

10. When was the last time you smoked?

☐ less than an hour ☐ 1-2 hours before

☐ more than 2 hours

11. When was the last time you consumed any meal?

☐ less than an hour ☐ 1-2 hours before

☐ more than 2 hours

12. When was the last time you drunk coffee?

☐ less than an hour ☐ 1-2 hours before

☐ more than 2 hours

13. When was the last time you had any oral hygiene procedure?

☐ less than an hour ☐ 1-2 hours before

☐ more than 2 hours

Medication and Suppliments

14. Do you use Ephedrine or any other energy boosters / weight cutters?

☐ Yes ☐ No

15. Do you use Protein or Creatine or any other Weight Gainers?

☐ Yes ☐ No

16. Do you use anabolic steroids or steroids of any sort?

☐ Yes ☐ No

17. Do you use any other hormones? (HGH, Insulin, Thyroxine, Etc)

☐ Yes ☐ No

18. Do you take anything to enhance recovery from training?

☐ Yes ☐ No

19. Have you taken ANY prescription medications or other substances in pas 3 months?

☐ Yes ☐ No

Statement of informed consent for participation

☐ I hereby consent to the processing of the personal data that I have provided and declare my agreement with the data protection regulations in the data privacy statement.

☐ I am fully aware of the implications of publication the research findings online in open access format and accept any associated risk.

Athlete signature

____
